# *FHL1* Downregulation Is Specific to Cancer-Associated Fibroblasts and Linked to Reduced Chromatin Accessibility in Colorectal Cancer

**DOI:** 10.3390/genes17101210

**Published:** 2026-09-29

**Authors:** Abdullah A. Alqasem, Waad Al-Burayk, Muneira Habjar, Batla Aldawsari, Alanoud Almansour, Mohammad A. Alahmari, Adel M. Alqarni, Helal G. Alanazi, Mohammed M. Alruwaili, Emad Alqassim, Ahmed Rokan, Abdulrahman A. Alahmari

**Affiliations:** 1 Department of Medical Laboratory, College of Applied Medical Sciences, Prince Sattam bin Abdulaziz University, Alkharj 11942, Saudi Arabia; a.alqasem@psau.edu.sa (A.A.A.); 444051457@std.psau.edu.sa (W.A.-B.); 444051650@std.psau.edu.sa (M.H.); 444051710@std.psau.edu.sa (B.A.); 443051351@std.psau.edu.sa (A.A.); a.alqarni@psau.edu.sa (A.M.A.); h.alanazi@psau.edu.sa (H.G.A.); a.rokan@psau.edu.sa (A.R.); 2 Laboratory Genetics Specialist, Forensic Medical Services Center, Ministry of Health, Riyadh 13211, Saudi Arabia; maalahmari@moh.gov.sa; 3 Department of Medical Laboratory Technology, College of Applied Medical Sciences, Northern Border University, Arar 91431, Saudi Arabia; mohammed.alruwaili@nbu.edu.sa; 4 Center for Health Research, Northern Border University, Arar 91431, Saudi Arabia; 5 Department of Anatomic Pathology, Pathology and Laboratory Medicine, King Faisal Specialist Hospital and Research Center, Riyadh 11211, Saudi Arabia; ealqassim@kfshrc.edu.sa; 6 Pathology Department, College of Medicine, Alfaisal University, Riyadh 11533, Saudi Arabia

**Keywords:** colorectal cancer, *FHL1*, cancer-associated fibroblasts, chromatin accessibility

## Abstract

Background/Objectives: Colorectal cancer (CRC) is among the most diagnosed malignancies and is a leading cause of cancer-related deaths globally. Beyond canonical CRC driver mutations, additional molecular alterations such as dysregulated gene expression and epigenetic reprogramming shape the disease behavior and may represent potential candidate biomarkers. The Four and a Half LIM domain (FHL) family, specifically *FHL1*, has been implicated as a tumor suppressor in CRC epithelial cells. However, *FHL1* expression is reduced in CRC and whether this reduction in heterogenous tumors is cell type-specific remains unknown. Methods: In this study, we integrated bulk genomic, transcriptomic and survival data from the cancer genome atlas (TCGA) with publicly available single-cell transcriptomic and epigenomic datasets to identify where and how *FHL1* expression is dysregulated in CRC. Results: While somatic mutations of FHL family genes are infrequent and non-recurrent, downregulation of *FHL1* in CRC tissue is evident and shows a modest association with longer survival that is not independent of clinical factors. Notably, single-cell analysis across three independent studies revealed that *FHL1* expression is largely restricted to fibroblasts and is specifically downregulated in cancer-associated fibroblasts (CAFs). *FHL1* expression is inversely associated with activated CAF markers FAP and *ACTA2*, while normal fibroblast markers *WNT2B* and *RSPO3* demonstrate a similar expression pattern to *FHL1*. Finally, chromatin accessibility at the *FHL1* promoter was substantially reduced in CAFs relative to pre-CAFs and normal fibroblasts. Conclusions: Our study identifies that *FHL1* downregulation is specific to CAFs in CRC and is accompanied by chromatin inaccessibility within the *FHL1* promoter in CAFs.

## 1. Introduction

Colorectal cancer (CRC) is the third most diagnosed cancer and the second leading cause of cancer-related deaths worldwide [1,2,3]. Despite improvements in CRC screening, diagnosis and treatment, a large proportion of patients experience disease progression or recurrence, suggesting an alternative molecular reprogramming that occurs as an adaptive mechanism in favor of the disease. CRC driver mutations such as *APC*, *KRAS* and *TP53* are well-characterized and their role in tumor initiation, progression and maintenance have been extensively investigated [4,5,6]. However, identification of subsequent molecular changes that may affect CRC behavior is a crucial need.

The Four and a Half LIM domain (FHL) family genes have emerged as regulators of tumor phenotype in multiple cancers [7]. At the protein level, these members function as adaptor proteins coupling the cytoskeletal network to intracellular signaling pathways [8,9,10]. Among the FHL genes, *FHL1* is the founding member and is physiologically important for muscle function [11,12]. The role of *FHL1* in cancer is complex where it acts predominantly as a tumor suppressor but can possess oncogenic activity in different cancers [13]. In CRC, *FHL1* has been shown to inhibit cancer cell proliferation and invasion through suppression of Wnt/β-catenin signaling programs [14]. Furthermore, *FHL1* has been shown to inhibit tumor growth in xenograft models [15]. Importantly, *FHL1* expression is consistently reduced in tumor tissues relative to normal colon and rectal tissues [14]. However, understanding where and how *FHL1* is downregulated in CRC remains an important question.

Prior studies have focused on transcriptomic analysis of *FHL1* from bulk RNA-sequencing datasets such as the cancer genome atlas (TCGA) [16]. These datasets measure gene expression from whole-tumor heterogeneous tissues containing not only epithelial cells but also other cell types such as immune and stromal cells. Therefore, interpreting bulk RNA expression is complicated further by the heterogeneity of the tumor stroma. Expression of genes mainly by stromal cells can therefore influence their associations with outcomes based on bulk expression data. Here, it is unclear whether *FHL1* expression is reduced in malignant epithelial cells specifically or other tumor microenvironment (TME) cell populations are involved. Additionally, it is not clear whether FHL genes, specifically *FHL1*, are frequently mutated or genetically silenced and therefore may directly affect gene expression. Therefore, cellular localization of *FHL1* loss and its mechanistic basis represent an important unresolved gap that can limit proper interpretation of *FHL1* potential as a cancer-related biomarker.

Here, we integrated bulk genomics, transcriptomics and survival data from TCGA with publicly available single-cell transcriptomics and epigenomics data to ask whether dysregulation of *FHL1* expression is cell type-specific, and if such dysregulation is associated with epigenetic reprogramming in CRC. We show that FHL gene mutations are infrequent and non-recurrent in CRC. However, FHL genes, specifically *FHL1*, are downregulated in tumor tissues, and low expression is associated with a modest favorable prognosis. At single-cell resolution, *FHL1* is expressed mainly by fibroblasts, and donor-level analysis shows that *FHL1* is consistently reduced in tumor-derived fibroblasts. Lastly, we reveal that chromatin accessibility at the *FHL1* promoter region is markedly reduced in CAFs compared to pre-CAFs and normal fibroblasts. Overall, our findings indicate that *FHL1* downregulation in CRC is largely a stromal event.

## 2. Materials and Methods

### 2.1. Analysis Cohort

The COAD and READ TCGA public somatic mutation data were obtained from the Genomic Data Commons (GDC) using the R package TCGAbiolinks (Version 2.32.0). Gene expression data from normal, adjacent normal and tumor tissues were retrieved from the TNMplot database (accessed on 15 April 2026) [17]. cBioPortal for cancer genomics (www.cbioportal.org) (accessed on 15 April 2026) [18,19] was used to download the survival database on quartile mRNA Expression, RSEM (Batch normalized from Illumina HiSeq_RNASeqV2) (accessed on 15 April 2026). Single-cell transcriptomic and epigenomic data were publicly available through the single-cell cancer explorer (scCancerExplorer; https://bianlab.cn/scCancerExplorer/explore/search) (accessed on 15 April 2026) [20]. For quantitative analysis, raw count matrices for EMTAB8107 were downloaded from ArrayExpress (accessed on 24 September 2026), while cell type annotations were obtained from the Tumor Immune Single Cell Hub 2 (TISCH2) database (accessed on 24 September 2026) [21]. For GSE132465, raw UMI counts and cell annotations were downloaded directly from GEO (accessed on 24 September 2026).

### 2.2. Somatic Mutation Analysis

Somatic mutation data for CRC was obtained from The Cancer Genome Atlas (TCGA) database using the R package TCGAbiolinks (Version 2.32.0). Two separate datasets were retrieved from the Genomic Data Commons (GDC): colon adenocarcinoma (TCGA-COAD) and rectal adenocarcinoma (TCGA-READ). Masked somatic mutation data were retrieved using the Aliquot Ensemble Somatic Variant Merging and Masking under the Simple Nucleotide Variation (SNV) category. Mutation annotation format (MAF) files from TCGA-COAD and TCGA-READ were merged and imported to maftools for further analysis using maftools R package (Version 2.20.0). The mutational landscape was plotted using oncoplot, visualizing the top ten mutated genes in CRC and the four FHL family genes. Samples with at least one alteration (n = 580) in the selected genes were displayed in the oncoplot. The distribution of mutation variants across different FHL gene domains was shown by lolliplot.

### 2.3. Bulk RNA-Seq Analysis

For mRNA expression of *FHL1* in CRC, data were first retrieved from the TNMplot database [17]. Log2-transformed expression values for FHL family genes were visualized using R with the tidyverse (Version 2.0.0) and ggplot2 (Version 4.0.2) packages. Expression values were plotted as a polar bar chart to illustrate differences in FHL gene expression between normal and tumor. For *FHL1* specifically, data was visualized and statistically compared using Prism Graphpad (10.3.1). The Mann–Whitney test was used to compare gene expression between normal and tumor tissues, while the Wilcoxon test was used to compare matched adjacent normal and tumor tissues. *p*-value < 0.05 was considered statistically significant.

### 2.4. Tissue Composition Association

To assess the relationship between bulk *FHL1* and tissue composition, stromal and smooth-muscle scores were calculated and correlated with *FHL1* using Spearman correlation. The stromal score was derived from the mean z-score of log2 expression of multiple stromal genes including COL1A1, COL1A2, COL3A1, DCN, LUM, PDGFRA, PDGFRB and *FAP*. The smooth-muscle score was derived from the mean z-score of log2 expression of DES, CNN1, MYH11 and ACTG2. *p* < 0.05 was considered statistically significant.

### 2.5. Single-Cell RNA-Seq Analysis

Single-cell (scRNA-seq) data were explored using the scCancerExplorer database (https://bianlab.cn/scCancerExplorer/explore/search) (accessed on 15 April 2026) [20]. This database is an integrated resource for interactive analysis of single-cell multi-omics across multiple cancers. Three scRNA-seq studies (HRA000201 [22], EMTAB8107 [23], and GSE132465 [24]) were analyzed with a focus on CRC samples in studies with multiple cancer types. The database processes scRNA-seq expression matrices using Seurat [25]. Gene expression was explored across annotated cell populations within each dataset. Expression distributions were visualized using UMAP and dot plots. Each dataset was examined independently to limit inconsistencies of cell type-specific expression patterns. Detailed description of specific preprocessing and analysis settings can be found in the original database study [20].

### 2.6. Single-Cell ATAC-Seq Analysis

Single-cell assay for transposase-accessible chromatin sequencing (scATAC-seq) data (GSE201349) [26] were explored using the single-cell epigenome browser module in the scCancerExplorer database. These data include samples from healthy colons, polyps, and CRC tissues. The data were processed using ArchR [27] by the database developers. Cells were selected based on quality control criteria reported in the original studies. In cases where such criteria were not available, cells with a TSS enrichment score more than or equal to 4 and mapped fragments of more than or equal to 1000 were retained. Identification and removal of cell doublets were performed using ArchR. Dimensionality reduction was performed using iterative latent semantic indexing and accessible chromatin coverage was identified by MACS2 [28]. Accessibility tracks were visually compared between selected annotated cell populations with a focus on regions within and surrounding the promoter and transcription start site (TSS). These comparisons are qualitative.

### 2.7. Donor-Level Pseudobulk Analysis of Single-Cell Data

Quantitative comparisons were performed at a donor-level using raw count matrices. For EMTAB8107, per-sample raw count matrices for the 21 CRC library (7 patients, each with tumor border, tumor core and adjacent normal tissues) were downloaded from ArrayExpress. Cell type annotations were obtained from the TISCH2 database [21]. Cells annotated as fibroblasts were included, and myofibroblasts were excluded because they were rare in tumor tissues. For GSE132465, raw UMI counts and cell annotations were downloaded directly from GEO. The fibroblast subtypes stromal 1, stromal 2, stromal 3 and myofibroblasts from normal and tumor tissues were analyzed. For each patient and tissue, raw counts were summed across fibroblasts to generate pseudobulk profiles (excluding profiles derived from less than 20 fibroblasts) as previously described [29]. Genes were filtered using filterByExpr and normalized by TMM (Trimmed Mean of M-values) within the edgeR package (Version 4.2.2) [30]. Limma-voom [31,32] with a paired design (~patient + tissue) were used to calculate differential expression and were restricted to patients with both normal and tumor profiles. In addition, cell-level mean expression and percentage of fibroblasts with non-zero expression were calculated, log-normalized and tested (comparing tumor to normal) using the two-sided exact paired Wilcoxon signed-rank test. Analyses are provided in Tables S2 and S3.

### 2.8. Survival Analysis

Progression-free survival (PFS) and disease-specific survival (DSS) data were downloaded from cBioPortal for cancer genomics (www.cbioportal.org) [18,19]. Patients were stratified into high (upper quartile) and low (lower quartile) *FHL1* mRNA expression. For multivariable analysis, the Cox proportional hazard model with *FHL1* as a continuous variable in all patients was used. Analysis was adjusted for age, sex, stage (AJCC pathologic stage) and tumor site (colon or rectum). Kaplan–Meier curves were generated using the “survival” (Version 3.8-6) and “survminer” (Version 0.5.2) R packages to determine the association between gene expression and clinical outcomes. The proportional hazard assumption was assessed with scaled Schoenfeld residuals, and a log-rank test was performed to compare survival outcomes between groups. *p*-value < 0.05 was considered statistically significant. All analyses performed in R used the R version 4.4.0.

## 3. Results

### 3.1. Mutational Landscape of FHL Family Genes in Colorectal Cancer

**Figure 1:**
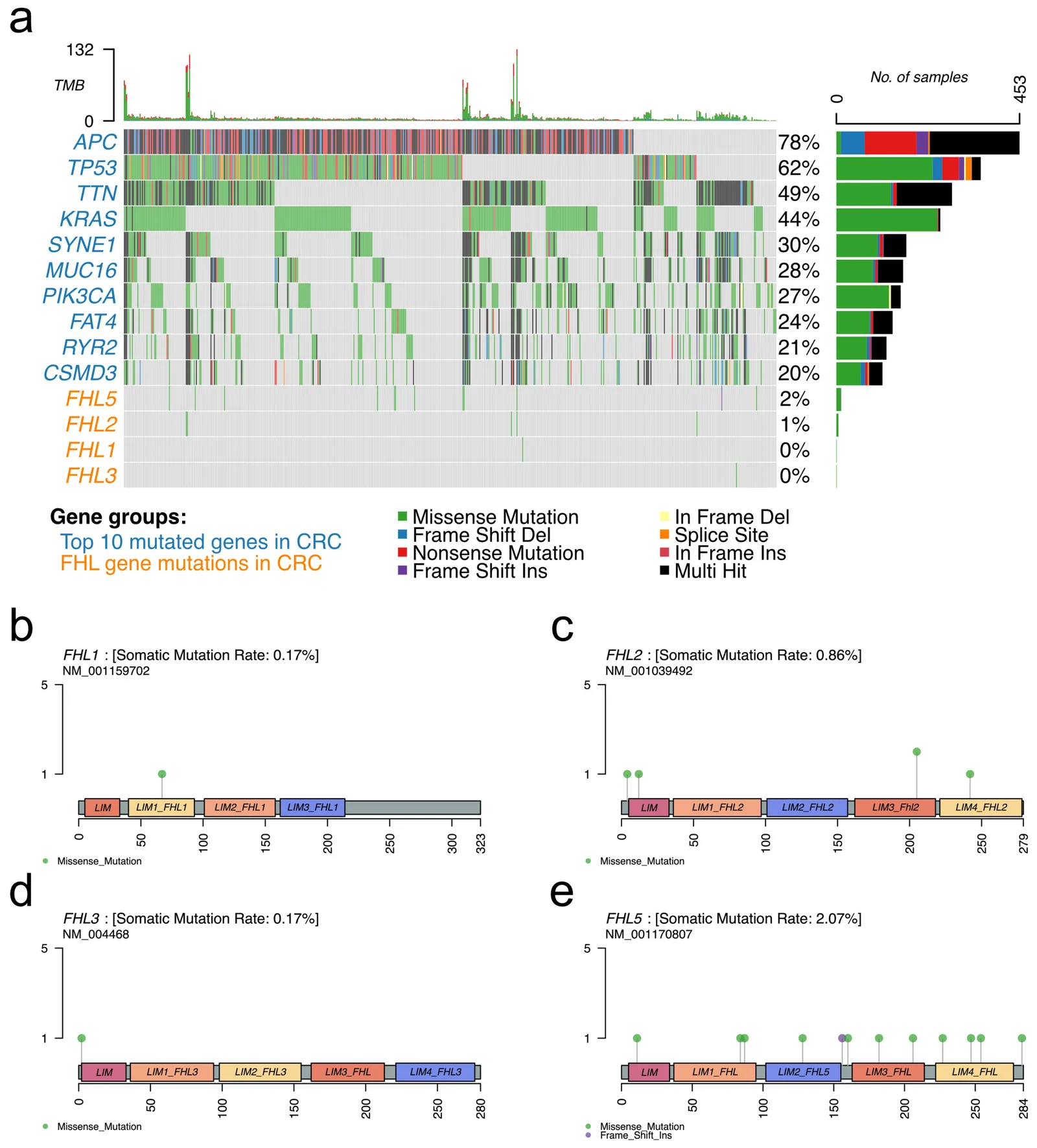
Mutational landscape of FHL family genes in CRC. (**a**) Oncoplot showing the mutational landscape of top 10 mutated genes (blue) and FHL family genes (orange) in CRC patients from TCGA. Percentages represent mutation rate relative to samples with at least one alteration in any of these gene. Types of mutations are color-coded below the oncoplot. (**b**–**e**) Lollipop plots showing the distribution of mutations across the *FHL1* (**b**), FHL2 (**c**), FHL3 (**d**) and FHL5 (**e**) gene domains. The Y-axis indicates the number of mutations for each specific variant.

To evaluate the somatic mutation landscape of the FHL gene family in CRC, we used the R package maftools to analyze whole-exome sequencing data from TCGA-COAD and TCGA-READ cohorts. Of these, only samples with at least one alteration in any selected gene were included in the oncoplot. FHL gene family members *FHL1*, FHL2, FHL3 and FHL5 were compared to the top ten mutated genes in CRC. In contrast to the canonical CRC genes, which are mutated at high frequency (Figure 1a, blue-labeled), the FHL genes were altered in only a small proportion of patients (Figure 1a, orange-labeled). *FHL1* and FHL3 show the lowest mutation frequency among FHL genes in CRC (<0.5% in *FHL1* and FHL3) (Figure 1a). FHL2 and FHL5 were mutated at a higher frequency than other FHL genes accounting for 1% and 2%, respectively (Figure 1a). To assess whether such infrequent mutations might nonetheless reflect a recurrent event, we used a lollipop plot to map the position of each variant onto its corresponding FHL protein domain (Figure 1b–e). *FHL1* and FHL3 each harbored a single missense mutation (somatic mutation rate of 0.17%) across all samples indicating no recurrent events (Figure 1b,d). The FHL2 somatic mutation rate was 0.86%, slightly higher than *FHL1* and FHL3 but still closely sparse. These mutations were dispersed across multiple domains with no two events occupying the same residue (Figure 1c). The most frequently mutated family member is FHL5 with a somatic mutation rate of 2.07% (Figure 1e). While FHL5 shows a broader spread of missense mutations across protein domains, there was no clustering pattern indicative of recurrent mutations. These data suggest that mutations of the FHL genes may not be biologically relevant.

### 3.2. FHL1 Expression Is Reduced in CRC and Shows a Modest Association with Patient Outcomes

**Figure 2:**
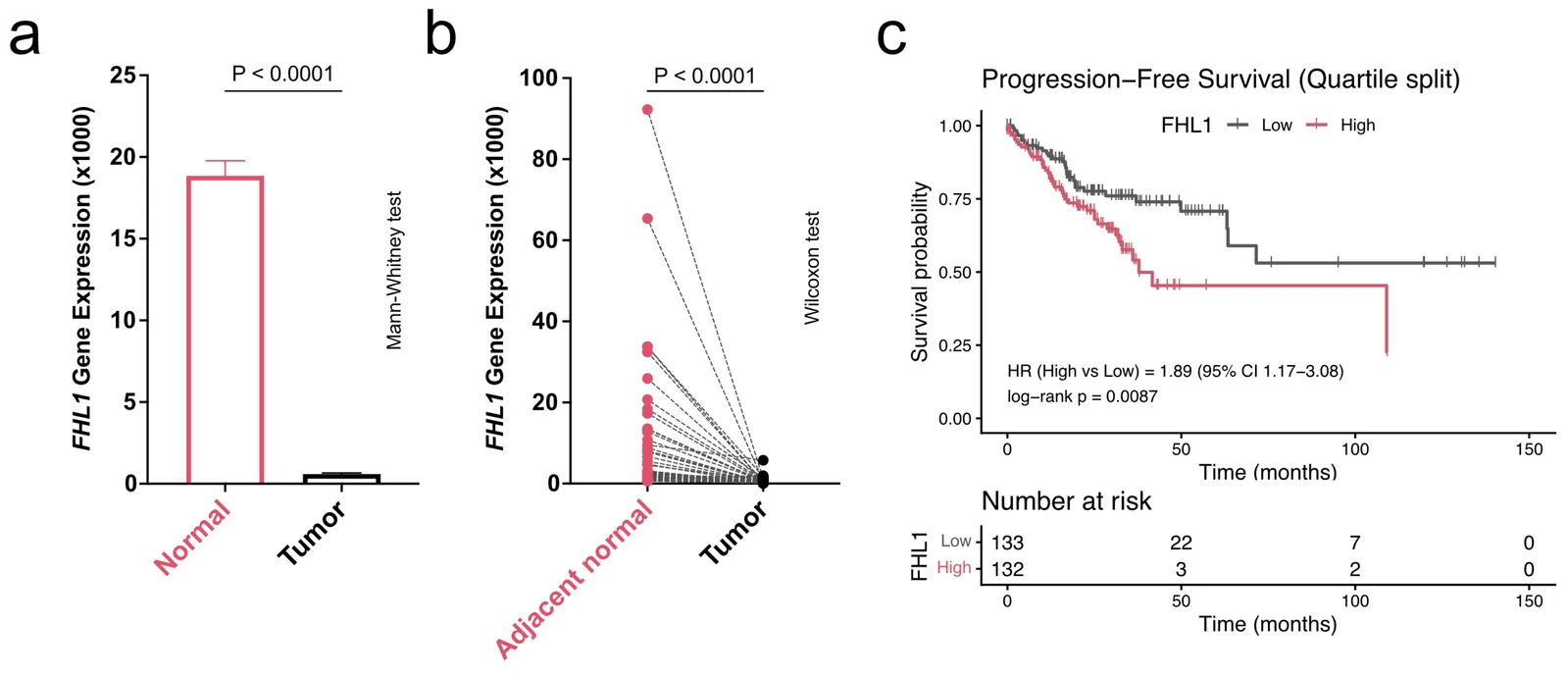
*FHL1* expression and progression-free survival in CRC. (**a**) Normalized expression values of *FHL1* in normal colon and CRC tumor tissues. Statistical comparison was performed using Mann–Whitney test (*p* < 0.0001). (**b**) Normalized expression values of *FHL1* in matched adjacent normal (non-malignant) and CRC tumor tissues. Statistical comparison was performed using Wilcoxon test (*p* < 0.0001). (**c**) Kaplan–Meier curves showing progression-free survival between high and low *FHL1* in CRC based on quartile cut-off. “Dark grey” indicates patients with low *FHL1* while “light red” indicates patients with high *FHL1* expression. The hazard ratio with 95% confidence interval and the log-rank *p*-value are shown. Tests were performed to compare survival outcomes between low and high *FHL1* groups using a univariable cox model without adjustment to clinical covariates.

We next sought to investigate whether expression of the FHL gene family is dysregulated in CRC by comparing gene expression profiles in tumor and non-tumor tissues. First, we examined the expression of all FHL members in tumor tissues from TCGA [16] and normal colon tissues from the Genotype-Tissue Expression (GTEx) [33] project. While FHL2 expression was slightly increased in tumor tissues, all other FHL family members had a lower expression in tumor tissues compared to normal colon tissues (Figure S1). This decrease was most pronounced for *FHL1*, which has previously been shown to inhibit proliferation of colorectal cancer cells [14]. Therefore, we focused our analysis on *FHL1* for its known biological relevance in CRC. To better compare the significant difference in *FHL1* expression between tumor and normal, we compared normalized expression values of *FHL1* in normal, adjacent normal, and tumor tissues. *FHL1* expression was significantly reduced in tumor tissues as compared to normal colon tissues (Mann–Whitney test, *p* < 0.0001) (Figure 2a). This finding was supported in a paired analysis of matched adjacent normal and tumor tissues, in which tumors had significantly lower *FHL1* expression than their paired normal (Wilcoxon test, *p* < 0.0001) (Figure 2b). Next, we sought to determine whether *FHL1* expression provides a prognostic value in CRC. We stratified CRC patients from TCGA into high and low *FHL1* expression groups based on the upper and lower quartile, respectively. Next, we performed Kaplan–Meier survival analysis using a univariable cox model and found that patients with low *FHL1* expression have a significantly longer progression-free survival (PFS) than those with high *FHL1* expression (HR = 1.89 [95% CI 1.17–3.08]; log-rank *p* = 0.0087) (Figure 2c). The corresponding difference in disease-specific survival (DSS) was trending toward improved outcomes although not statistically significant (HR = 1.91 [95% CI 0.93–3.90]; log-rank *p* = 0.071) (Figure S2). With *FHL1* modeled as a continuous variable in all patients, higher expression was associated with modestly shorter progression-free survival (HR: 1.2; 95% CI 1.02–1.42; *p* = 0.029) and disease-specific survival (HR: 1.28; 95% CI 1.00–1.64; *p* = 0.047) (Table S1). After adjustment for clinical covariates (age, sex, stage and tumor site), these associations were attenuated (Table S1). However, the adjusted quartile PFS remained significant (HR: 1.84; 95% CI 1.09–3.10; *p* = 0.022). Overall, continuous analyses suggest that this association is not completely independent of clinical factors.

### 3.3. FHL1 Is Expressed Mainly by Fibroblasts and Is Reduced in Cancer-Associated Fibroblasts

**Figure 3:**
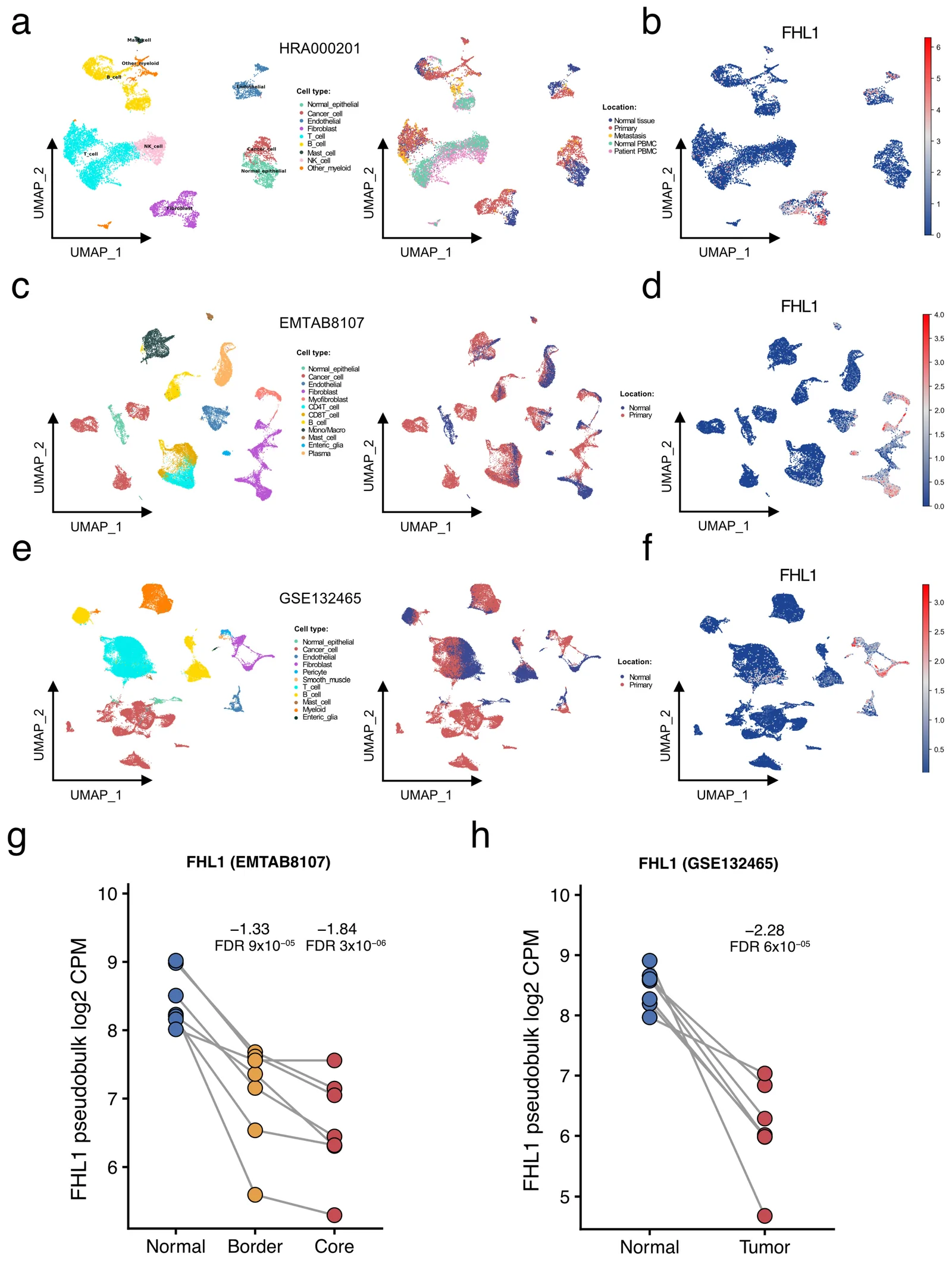
Single-cell transcriptomic analysis of *FHL1* in CRC tumor microenvironment. (**a**) UMAP clustering plots of the HRA000201 dataset showing cell type clusters (left) and cell location (right). (**b**) UMAP plots colored by *FHL1* expression for the HRA000201 dataset. (**c**) UMAP clustering plots of the EMTAB8107 dataset showing cell type clusters (left) and cell location (right). (**d**) UMAP plots colored by *FHL1* expression for the EMTAB8107 dataset. (**e**) UMAP clustering plots of the GSE132465 dataset showing cell type clusters (left) and cell location (right). (**f**) UMAP plots colored by *FHL1* expression for the GSE132465 dataset. (**g**,**h**) Donor-level pseudobulk analysis of *FHL1* expression in fibroblasts in EMTAB8107 (**g**) and GSE132465 (**h**).

Because *FHL1* is highly expressed in muscle cells [11,12], we asked whether bulk *FHL1* levels reflect tissue composition. *FHL1* expression was significantly correlated with the smooth-muscle score (Spearman rho = 0.81; *p* < 2.2 × 10^−16^) and with the stromal fibroblast score (Spearman rho = 0.63; *p* < 2.2 × 10^−16^) (Figure S3). Therefore, *FHL1* levels depend strongly on cellular composition, suggesting an investigation of *FHL1* expression at a single-cell resolution. We next asked whether downregulation of *FHL1* in CRC tumors is cell type-specific. We explored three independent publicly available single-cell RNA-seq (scRNA-seq) datasets. First, in the HRA000201 [22] dataset, cells were clustered into nine cell populations including epithelial cells, cancer cells, endothelial cells, fibroblasts, T cells, B cells, mast cells, natural killer [NK] cells and other myeloid cells (Figure 3a; left panel). These cells were sampled from normal tissues, primary tumors, metastasis and PBMC from normal individuals and cancer patients (Figure 3a; right panel). Intriguingly, *FHL1* expression was mainly restricted to the fibroblast cluster, with a negligible signal in other cell clusters (Figure 3a; left panel, Figure 3b). In addition, *FHL1* expression was markedly higher in normal fibroblasts and is clearly downregulated in CAF clusters (Figure 3a; right panel, Figure 3b). To further confirm these findings, we explored two additional scRNA-seq datasets from independent patient cohorts. These datasets were part of pan-cancer projects, but only CRC samples were analyzed here. In the EMTAB8107 [23] dataset, 14 tumor samples (seven from tumor border; seven from tumor core) and seven matched non-malignant (normal) samples from seven CRC patients were analyzed. Cells were clustered into many cell populations including normal epithelial cells, cancer cells, endothelial cells, fibroblasts, myofibroblasts, CD4+ and CD8+ T cells, B cells, monocytes and macrophages, mast cells, enteric glia, and plasma cells (Figure 3c; left panel). Similar to the first dataset, *FHL1* expression was confined almost exclusively to fibroblasts (Figure 3c; left panel, Figure 3d). The expression was higher in the region of the cluster enriched for normal fibroblasts and markedly reduced in the CAF region of the cluster (Figure 3c; right panel, Figure 3d). In the GSE132465 [24] dataset, profiled primary CRC (n = 23) and matched normal samples (n = 10/23) had multiple clusters comprising normal epithelial cells, cancer cells, endothelial cells, fibroblasts, pericytes, smooth muscle cells, T cells, B cells, mast cells, myeloid cells and enteric glia cell populations (Figure 3e; left panel). *FHL1* expression again localized to the fibroblast cluster and was similarly reduced in CAFs relative to fibroblasts derived from normal regions (Figure 3e; right panel, Figure 3f). Since these observations were based on expression from pooled cells across patients, we quantified *FHL1* expression in each patient using fibroblast pseudobulk profiles (refer to Section 2 for details). In EMTAB8107, *FHL1* expression was significantly lower in tumor-derived than in normal-derived fibroblasts (Figure 3g; Table S2). This reduction was greater in the tumor core than at the tumor border (tumor border log2FC −1.33, 95% CI −1.74 to −0.91; tumor core log2FC −1.84, 95% CI −2.28 to −1.41) (Figure 3g; Table S2). Similarly, in GSE132465 (only seven patients had sufficient fibroblasts in both normal and tumor tissues), *FHL1* expression was reduced in tumor-derived rather than in normal-derived fibroblasts (log2FC −2.28; 95% CI −2.85 to −1.71) (Figure 3h; Table S3).

Because of the heterogeneity nature of fibroblasts, we asked if *FHL1* reduction reflects downregulation within fibroblast subtypes using available annotations for GSE132465. In normal tissues, fibroblasts consisted almost entirely of stromal cells (stromal 1–3; Figure S4a). In tumor tissues, however, the fibroblast compartment was dominated by myofibroblasts (Figure S4a). *FHL1* expression was higher in normal stromal 1 and stromal 3 cells compared to stromal 2 (Figure S4b, colored in pink). However, the only subtype that is well-represented in both normal and tumor tissues is the stromal 2 cell population as other stromal subtypes were too scarce in tumors. Stromal 2 cells showed similar *FHL1* levels in normal and tumor tissues (Figure S4b). By comparing fibroblasts subtypes within tumor tissues, *FHL1* expression was lower in myofibroblasts compared to stromal 2 cell populations (Figure S4b). Therefore, *FHL1* downregulation in tumor fibroblasts largely reflects a shift from the *FHL1*-high stromal subtype toward the *FHL1*-low myofibroblast subtype.

### 3.4. FHL1 Expression Is Inversely Associated with Activated CAF Markers in Fibroblasts

**Figure 4:**
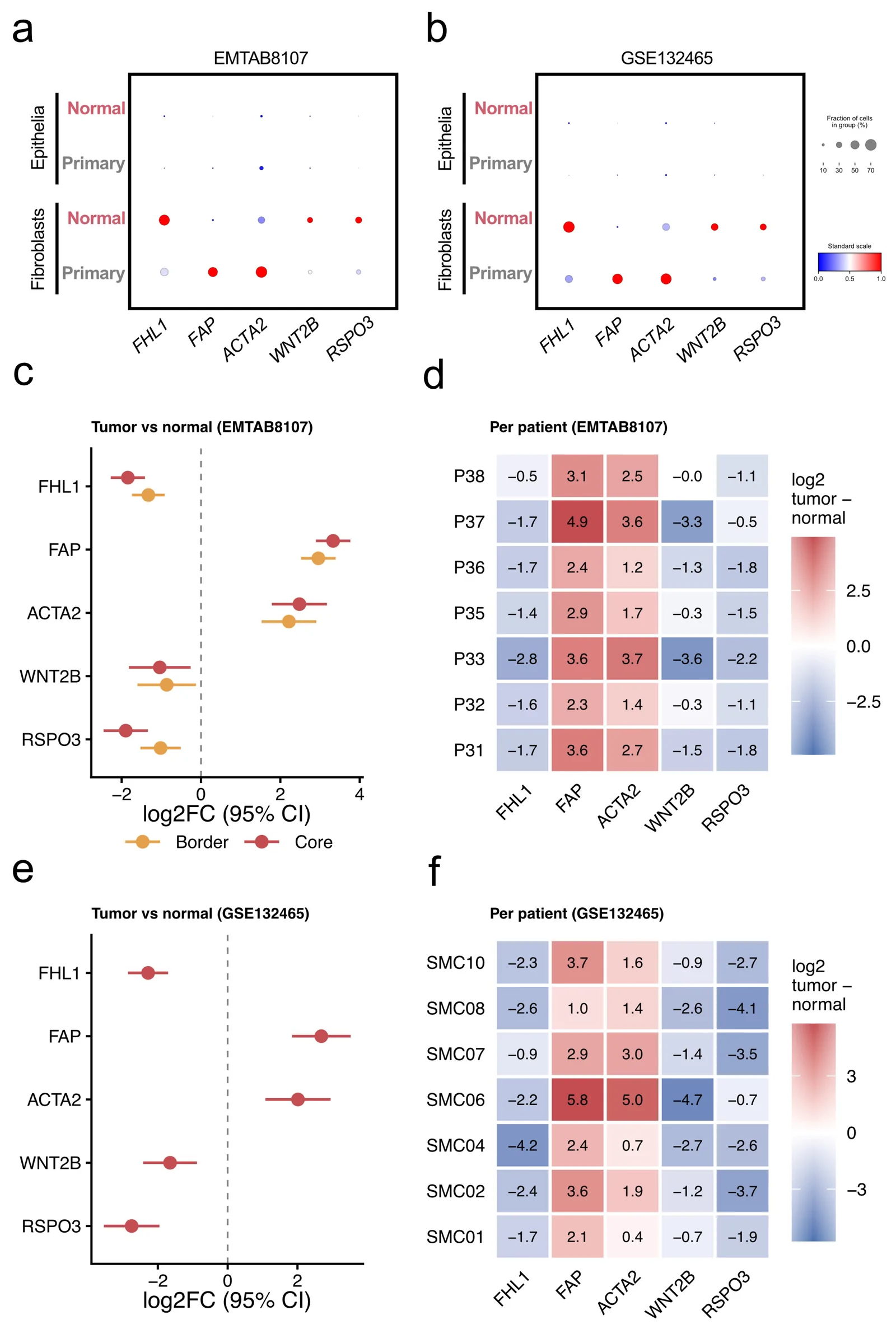
Expression of *FHL1* in relation to fibroblast subtype markers in CRC. (**a**,**b**) Dot plots showing the expression of *FHL1*, the CAF markers *FAP* and *ACTA2*, and the normal fibroblast markers *WNT2B* and *RSPO3* in (**a**) EMTAB8107 and (**b**) GSE132465 datasets. Dot size indicates the fraction of cells expressing each gene; color indicates standardized mean expression. (**c**,**d**) Donor-level pseudobulk analysis of fibroblasts in EMTAB8107, with log2 fold changes (95% CI) from tumor border and tumor core versus normal (**c**) and per-patient change in pseudobulk expression (**d**). (**e**,**f**) Donor-level pseudobulk analysis of fibroblasts in GSE132465, with log2 fold changes (95% CI) from tumor versus normal (**e**) and per-patient change in pseudobulk expression (**f**).

To place CAF-specific downregulation of *FHL1* in the context of well-established fibroblast markers, we compared *FHL1* expression to markers of normal fibroblasts and activated CAFs across epithelial and fibroblast cell populations. Two markers of activated CAFs, Fibroblast Activation Protein (*FAP*) and Alpha-Smooth Muscle Actin (a-SMA, also known as *ACTA2*), were explored [26]. For normal fibroblasts, Wnt Family Member 2B (*WNT2B*) and R-Spondin 3 (*RSPO3*) markers were examined [26]. Across all three datasets (HRA000201 [22], EMTAB8107 [23], and GSE132465 [24]), epithelial cells showed minimal expression of all markers as well as *FHL1,* suggesting that they are more associated with non-epithelial cell populations (Figure 4a,b and Figure S5). Within the fibroblast cell population, a consistent but reciprocal expression pattern was observed between *FHL1* and the CAF markers *FAP* and *ACTA2* (Figure 4a,b and Figure S5). In normal fibroblasts, *FHL1* was highly expressed, while CAF markers *FAP* and *ACTA2* were expressed at low levels. In primary tumor fibroblasts, a completely reverse expression was observed where *FHL1* expression is reduced as compared to *FAP* and *ACTA2*, for which expression is drastically increased (Figure 4a,b and Figure S5). This inverse relationship was reproduced in all three explored datasets. In contrast, the expression of normal fibroblast markers *WNT2B* and *RSPO3* was consistent with the same expression pattern of *FHL1*. Expression of *FHL1*, *WNT2B* and *RSPO3* was higher in normal fibroblasts and markedly reduced in CAFs.

Pseudobulk analysis in EMTAB8107 and GSE132465 confirmed this pattern in patients at an individual level. In both cohorts, *FAP* and *ACTA2* were increased while *WNT2B* and *RSPO3* was decreased in tumor-derived fibroblasts in every patient (Figure 4c–f and Figure S6; Tables S2 and S3). For example, in EMTAB8107, *FAP* increased by 2.96 log2FC at the tumor border and 3.33 in the tumor core (Figure 4c; Table S2). Collectively, these data confirm the finding that *FHL1* downregulation is more specific to CAFs than other cell populations within CRC tumors.

### 3.5. Reduced Chromatin Accessibility at the FHL1 Promoter Accompanies Fibroblast Activation

**Figure 5:**
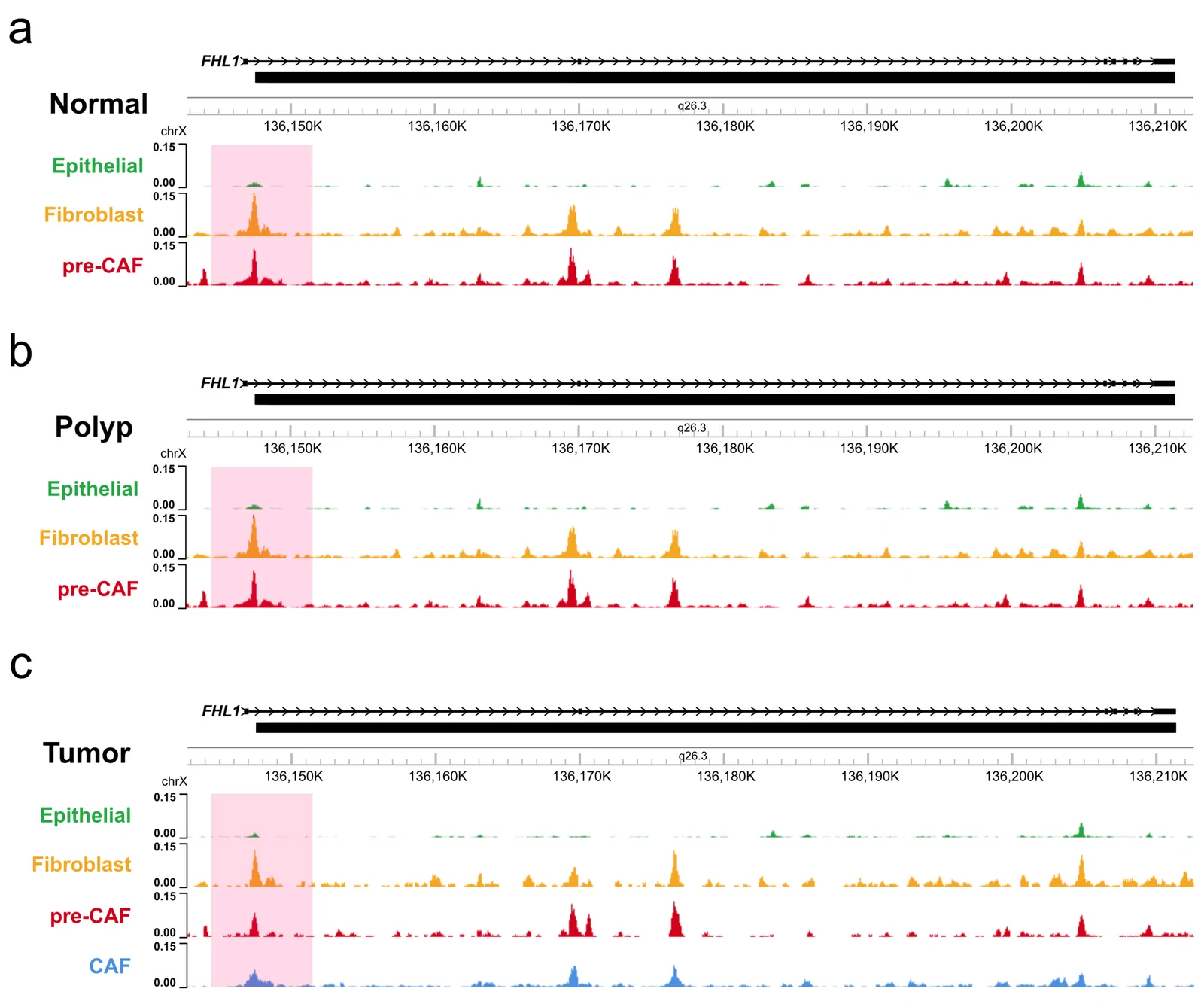
Chromatin accessibility at the *FHL1* locus in CRC fibroblast populations. (**a**–**c**) Genome browser tracks of single-cell chromatin accessibility (GSE201349 scATAC-seq dataset) at the *FHL1* locus in (**a**) normal colon, (**b**) polyps, and (**c**) tumor colorectal tissues. Coverage tracks are color-coded where “green” denotes epithelial, “orange” denotes fibroblasts, “red” denotes pre-CAFs and “blue” denotes tumor CAFs. The *FHL1* promoter is highlighted in pink. All tracks were plotted on a unified y-axis scale for reliable comparison.

To assess whether *FHL1* downregulation in CAFs is accompanied by changes in chromatin accessibility at the *FHL1* promoter, we explored scATAC-seq data of healthy colon, polyps and CRC (GSE201349) [26]. We then examined chromatin accessibility coverage at the promoter region of *FHL1* across epithelial, normal fibroblast, pre-CAF and CAF cell populations (Figure 5). In normal tissues, the promoter region was more accessible in fibroblasts and pre-CAFs compared to epithelial tissues (Figure 5a). Similarly, polyp tissues have more chromatin accessibility at the *FHL1* promoter in fibroblasts and pre-CAFs compared to epithelial tissues (Figure 5b). In both normal colon and polyps, accessibility was less pronounced in pre-CAFs compared to normal fibroblasts (Figure 5a,b, orange and red tracks). In CRC tissues, chromatin accessibility decreases as fibroblast subtypes changes from fibroblast to pre-CAF and finally to CAFs, with CAFs having the least chromatin accessibility at the *FHL1* promoter (Figure 5c). Of note, chromatin accessibility coverage at the *FHL1* promoter in epithelial tissues did not change between normal colon, polyps and CRC (Figure 5a–c, green tracks). In contrast, fibroblasts and pre-CAFs from CRC tissues showed lower accessibility than the corresponding populations from normal colon and polyps (Figure 5a–c, orange and red tracks). Reduced accessibility at the FHL1 promoter is therefore a feature of fibroblast populations in tumor tissues, most pronounced in CAFs, and is not observed in epithelial cells. Taken together, these findings may suggest that the closed chromatin structure in CAFs contributes to restricted transcriptional activity of *FHL1* in CRC.

## 4. Discussion

Our study advances the understanding of *FHL1* dysregulation in CRC in several ways. First, while previous studies established *FHL1* as a tumor suppressor in CRC cells, those studies used data from bulk tumor tissues [14]. Therefore, which cells within such a heterogenous tumor microenvironment contribute to *FHL1* loss was not resolved. Second, whether *FHL1* downregulation is driven by recurrent genetic alteration or by another regulatory molecular mechanisms had not been investigated. Third, the link between *FHL1* expression and the chromatin state had not been established. To our knowledge, our study is the first to integrate bulk genomics, transcriptomics, single-cell transcriptomics and epigenomics to localize *FHL1* downregulation within the CRC tumor microenvironment. We show that *FHL1* somatic mutations are infrequent and non-recurrent. We show that *FHL1* is downregulated in tumor tissues and its reduction is associated with modest better outcomes in CRC patients. Notably, we discovered that *FHL1* downregulation is specific to CAFs, and that loss of *FHL1* expression is accompanied by lower chromatin accessibility at the *FHL1* regulatory regions.

Genetic mutations can inactivate tumor suppressor genes, thus reshaping tumor behavior. *FHL1* has been shown to have an inactivating mutation of its tumor-suppressing activity in gastrointestinal cancers [15]. This raised the question of whether *FHL1* harbors recurrent mutations that may be biologically relevant in CRC and may affect *FHL1* expression. However, we found that *FHL1*, and other FHL family genes, are mutated at a very low frequency. These observed infrequent variants were also dispersed across protein domains indicating no recurrent events. These data indicate that mutational events are unlikely to account for *FHL1* dysregulation in CRC and point instead to other non-genetic regulatory mechanisms. In line with this, previous reports have shown that *FHL1* can be silenced post-transcriptionally by miR-410 to eventually enhance tumor growth [34]. Our data support the notion that dysregulated *FHL1* expression arises from regulatory rather than mutational events in CRC.

*FHL1* inhibits proliferation and invasion of CRC cells in vitro and tumor growth in xenograft models and is therefore regarded as a tumor suppressor gene [14,15,34]. However, our analysis showed that low *FHL1* expression is modestly associated with favorable progression-free survival in CRC patients. This association was significant in unadjusted continuous analyses based on the quartile cut-off, and it was attenuated when adjusted for clinical covariates in continuous models. This inconsistency in predicting patient outcomes may be explained by our single-cell analysis. *FHL1* is expressed mainly by fibroblasts rather than tumor epithelium, and it is highly expressed in muscle cells [11,12]. Consistent with this, we showed that bulk *FHL1* expression is strongly correlated with muscle and stromal content. Muscle and stromal contents varies with tumor heterogeneity, and stromal content has been shown to predict prognosis in CRC [35,36]. The direction and strength of bulk FHL1 outcome associations may therefore differ between studies for reasons unrelated to FHL1 regulation within any single cell type. This importantly underscores the limitation of bulk profiling of gene expression as well as targeting *FHL1* selectively in cancer epithelial cells. It is therefore reasonable to hypothesize that *FHL1* expression represents an aggregate signal not only from malignant epithelial cells but also from diverse tumor microenvironmental cell populations. We here found that *FHL1* is predominantly expressed in fibroblasts rather than epithelial cells. We found that *FHL1* expression is drastically reduced in CAFs as compared to normal fibroblasts, indicating that bulk *FHL1* levels largely reflect the CRC stromal microenvironment. This is consistent with prior reports showing that expression of another FHL family member, *FHL2*, is specific to fibroblast populations in lung cancer [37,38]. Importantly, *FHL2* loss in CAFs decreases CAF-induced migration and invasion of human lung adenocarcinoma cells in vitro and metastasis in vivo [38]. Together, these findings indicate that *FHL1* is more specific to fibroblasts and suggest that *FHL1* may possess a biological role in CRC in a CAF-dependent manner.

CAFs are a heterogenous population that arises from multiple cellular origins and adopts distinct activation states within the TME [39,40]. Our central finding is that *FHL1* downregulation is specific to CAFs in CRC. Across all three studies analyzed, *FHL1* expression was confined almost exclusively to the fibroblast clusters and was substantially reduced in CAFs relative to fibroblasts from normal tissues or from adjacent non-malignant tissues. Donor-level analysis showed that *FHL1* is reduced in tumor-derived compared to normal-derived fibroblasts in every patient of two independent cohorts. Subtype analysis showed that this reduction largely reflects a change in fibroblast composition where high *FHL1* stromal contents are replaced with low *FHL1* myofibroblasts in tumors. Consistent with fibroblast transition from a quiescent to an activated state, *FHL1* was inversely expressed compared to the activated CAF markers *FAP* and *ACTA2*. In contrast, *FHL1* expression was concordant with the normal fibroblast markers *WNT2B* and *RSPO3*. These data may position *FHL1* as marker of normal non-activated fibroblasts that is lost as fibroblasts transition to CAFs. The structure of the *FHL1* protein domain may provide a possible mechanistic link with fibroblast function. The LIM domains of *FHL1*, as for other FHL family members, can sense mechanical strains by directly binding to tensed actin filaments [41]. During CAF activation, fibroblasts undergo increased contractility and cytoskeletal and extracellular matrix (ECM) remodeling [42]. Therefore, it is likely that the loss of *FHL1* during this transition may be functionally connected to the acquisition of an activated fibroblast state.

*FHL1* is located on the X chromosome and therefore can be affected by a single-hit due to chromosome X distinct methylation patters. To better understand CAF-specific *FHL1* loss, we examined chromatin accessibility at the *FHL1* locus and found that the promoter region was inaccessible in CAFs compared to pre-CAFs and normal fibroblasts. Chromatin accessibility at regulatory regions, especially promoters, is a principal determinant of transcriptional activity, with decreased accessibility being frequently associated with gene silencing [43]. The concordance between reduced promoter accessibility and *FHL1* expression in CAFs is compatible with *FHL1* silencing through closure of promoter regulatory elements during CAF activation, although our data cannot determine whether reduced accessibility is a cause or a consequence of *FHL1* downregulation. The fact that *FHL1* promoter accessibility in epithelial cells did not change between normal colon, polyps and tumors indicates that this epigenetic reprogramming is a stromal rather than an epithelial event. This is consistent with previous reports that CAF activation is driven in part by epigenetic remodeling [44] and that *FHL1* in CAFs is subject to epigenetic silencing in the gastrointestinal tract [15]. Whether a closed-chromatin state at the *FHL1* regulatory regions, including promoter, is required for *FHL1* silencing in CAFs remains to be determined.

There are several limitations to this study that warrant further investigation. First, our analyses rely on publicly available datasets and the findings here are correlative rather than experimentally validated. Second, the chromatin accessibility comparisons are qualitative based on aggregated coverage tracks and therefore do not establish causality. Validation with complementary approaches such as bisulfite sequencing under induced-fibroblast activation settings is needed. Third, whether *FHL1* reduction in CAFs is a cause or a consequence of CAF activation is not addressed in this study. Future studies, such as gain- or loss-of-function experiments in fibroblasts, need to investigate the functional consequence of *FHL1* loss on CAF behaviors. Fourth, survival analyses were based on bulk tumor expression of *FHL1*, which is influenced by tissue composition. Finally, our analysis is CRC-focused and whether CAF-specific *FHL1* findings generalize to other cancer types remains an open question.

In conclusion, our integrative multi-omics analysis identifies *FHL1* loss in CRC as a cell type-specific event that preferentially occurs in activated tumor CAFs in comparison with pre-CAFs and inactivated normal fibroblasts. This *FHL1* loss is associated with an inaccessible *FHL1* promoter in CAFs but not epithelial cells. *FHL1* expression from bulk tumors shows a modest association with patient outcomes. Therefore, our data caution against relying on bulk *FHL1* expression without regard for the cellular context especially when targeting *FHL1* in only epithelial cells. Future work to dissect the epigenetic control of *FHL1* in CAFs is needed to determine whether *FHL1* represents a potential stromal biomarker or a therapeutic vulnerability in CRC.

## Data Availability

The original data presented in the study are openly available through the following databases: the Genomic Data Commons (GDC) (https://gdc.cancer.gov/about-data/publications/pancanatlas) (accessed on 15 April 2026), cBioPortal for cancer genomics (www.cbioportal.org) (accessed on 15 April 2026), and the scCancerExplorer database (https://bianlab.cn/scCancerExplorer/explore/search) (accessed on 15 April 2026). The sequence data for HRA000201 is deposited in the Genome Sequence Archive, National Genomics Data Center, Beijing Institute of Genomics (China National Center for Bioinformation), Chinese Academy of Sciences, and is publicly accessible at http://bigd.big.ac.cn/gsa-human (accessed on 15 April 2026). The EMTAB8107 (accessed on 15 April 2026) dataset is available through the EMBL-EBI ArrayExpress collection (https://www.ebi.ac.uk) (accessed on 15 April 2026) under accession number E-MTAB-8107. The GSE132465 (accessed on 24 September 2026) and GSE201349 (accessed on 15 April 2026) are available through the NCBI Gene Expression Omnibus (GEO) (https://www.ncbi.nlm.nih.gov/geo/) (accessed on 15 April 2026). Codes used in this study, in general, were not original and were used from publicly available R packages described in the Section 2. Other custom scripts are available from the corresponding author on request.

## Supplementary Materials

The following supporting information can be downloaded at: https://www.mdpi.com/article/10.3390/genes17101210/s1, Figure S1: Polar plot of log2 transformed expression of FHL family genes in normal (GTEx; light red) and tumor (TCGA; dark grey) tissues. Figure S2: Kaplan-Meier curves showing disease-specific survival between high and low FHL1 in CRC. “Dark grey” indicates patients with low FHL1 (lower quartile) while “light red” indicates patients with high FHL1 expression (top quartile). A log-rank test was performed to compare survival outcomes between low and high FHL1 groups without adjustment to clinical covariates. Figure S3: Correlation of bulk FHL1 expression (log2 RSEM + 1) with (a) the smooth-muscle score and (b) the stromal fibroblast score in the TCGA CRC cohort. Spearman correlation coefficients and P values are shown. Figure S4: Fibroblast subtypes in GSE132465. (a) Fibroblast subtype composition (Stromal 1, Stromal 2, Stromal 3, myofibroblasts) in normal and tumor tissue of each patient. (b) Mean log-normalized FHL1 expression by fibroblast subtype in normal and tumor tissue; each point is one patient, and point size indicates the number of cells. Figure S5: Dot plot of FHL1, FAP, ACTA2, WNT2B and RSPO3 in epithelial cells and fibroblasts from normal and primary tumor tissue in HRA000201. Dot size indicates the fraction of cells expressing each gene; color indicates standardized mean expression. Figure S6. Donor-level mean expression (log-normalized) of FHL1, FAP, ACTA2, WNT2B and RSPO3 in fibroblasts from (a) normal tissue, tumor border and tumor core in EMTAB8107 and (b) normal and tumor tissue in GSE132465. Each point is one patient; point size indicates the number of fibroblasts, and lines connect samples from the same patient. Table S1: Multivariable analysis of FHL1 in CRC. Table S2: Donor-level pseudobulk analysis of EMTAB8107. Table S3: Donor-level pseudobulk analysis of GSE132465.

## Abbreviations

The following abbreviations are used in this manuscript:

*ACTA2*	Alpha-Smooth Muscle Actin
ATAC-seq	Assay for transposase-accessible chromatin sequencing
CAF	Cancer-associated fibroblast
COAD	Colon adenocarcinoma
CRC	Colorectal cancer
DSS	Disease-specific survival
*FAP*	Fibroblast Activation Protein
FHL	Four and a Half LIM domain
GTEx	Genotype-Tissue Expression
PFS	Progression-free survival
READ	Rectal adenocarcinoma
RNA-seq	RNA sequencing
*RSPO3*	R-Spondin 3
TCGA	The Cancer Genome Atlas
TME	Tumor microenvironment
TSS	Transcription start site
*WNT2B*	Wnt Family Member 2B
