# Supplementary material for: FHL1 Downregulation Is Specific to Cancer-Associated Fibroblasts and Linked to Reduced Chromatin Accessibility in Colorectal Cancer
Source: Genes (Basel). 2026 Sep 29;17(10):1210. doi: 10.3390/genes17101210 (PMC13704643; doi:10.3390/genes17101210)
Supplement: Supplementary file 1 [file genes-17-01210-s001.zip › Supplementary Figures.pdf]

Figure S1

a

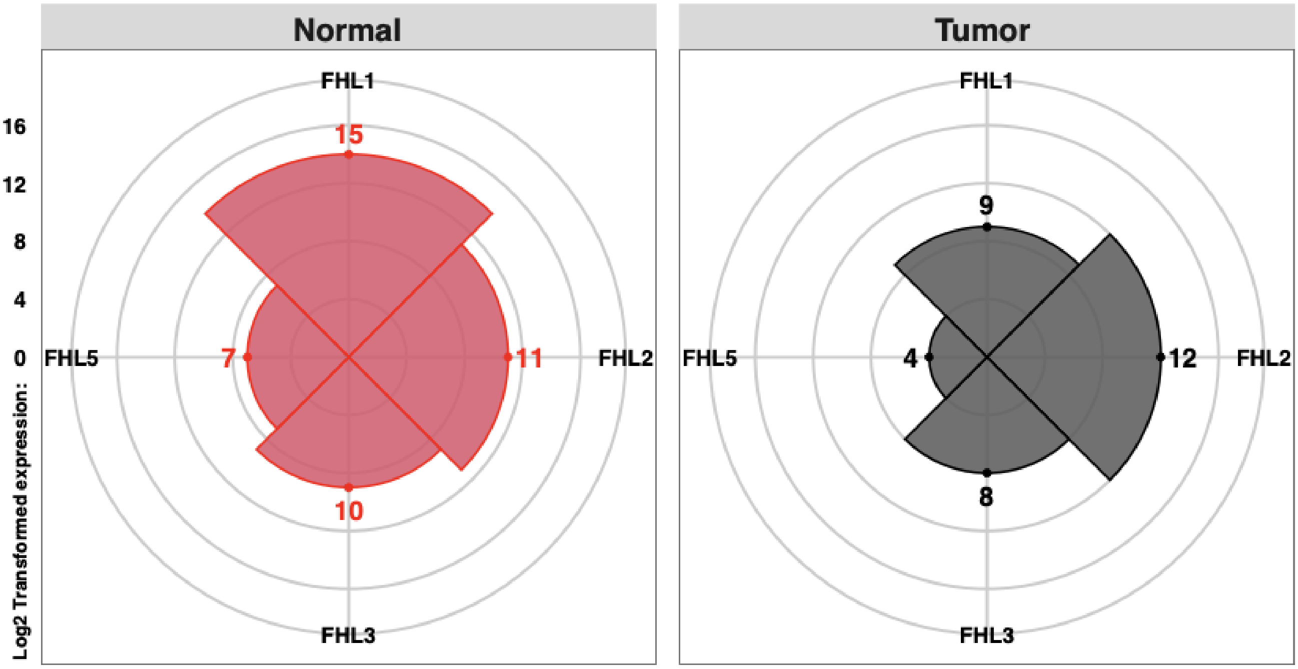

Figure S1. Polar plot of log2 transformed expression of FHL family genes in normal (GTEx; light red) and tumor (TCGA; dark grey) tissues.

Figure S2

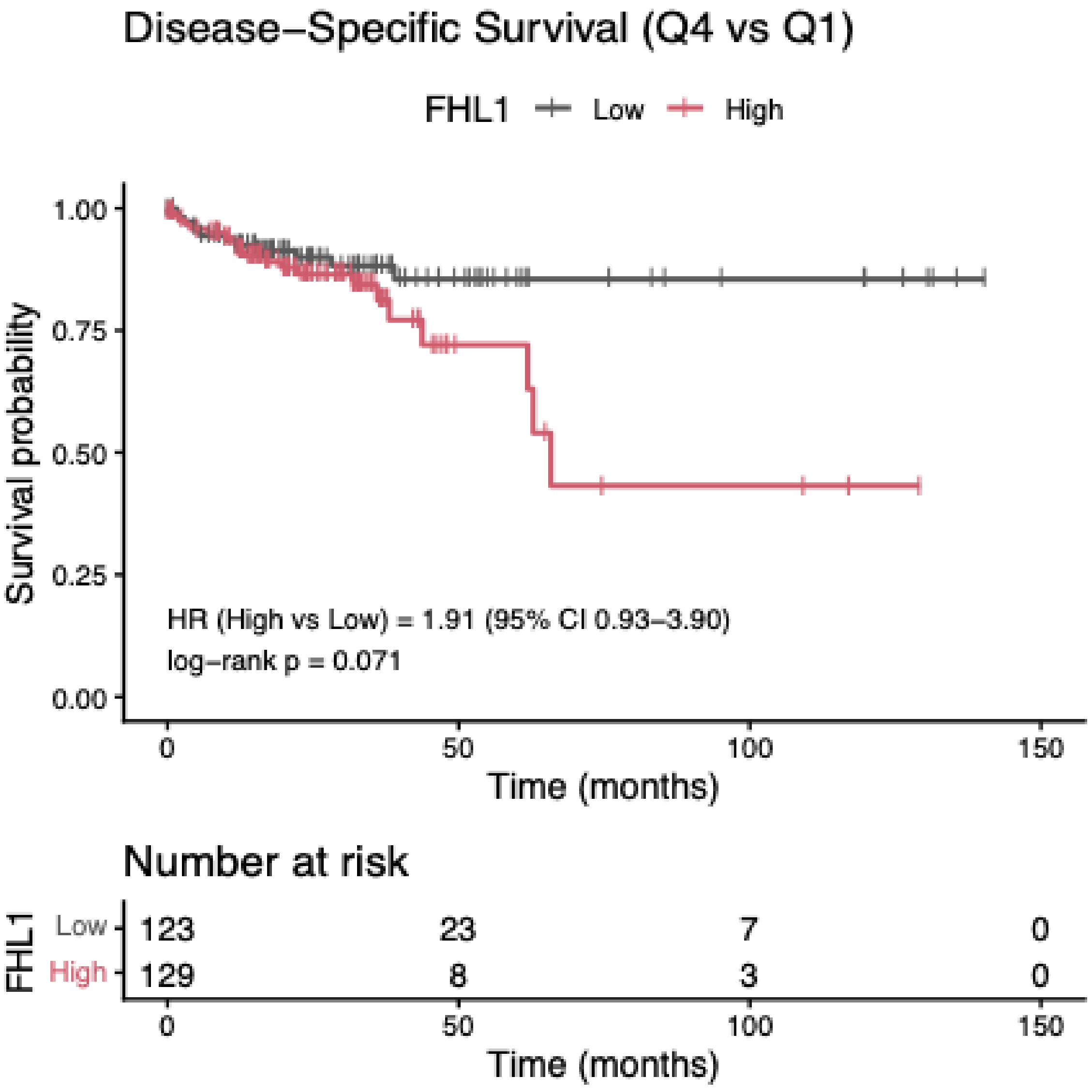

Figure S2. Kaplan-Meier curves showing disease-specific survival between high and low FHL1 in CRC. "Dark grey" indicates patients with low FHL1 (lower quartile) while "light red" indicates patients with high FHL1 expression (top quartile). A log-rank test was performed to compare survival outcomes between low and high FHL1 groups without adjustment to clinical covariates.

Figure S3

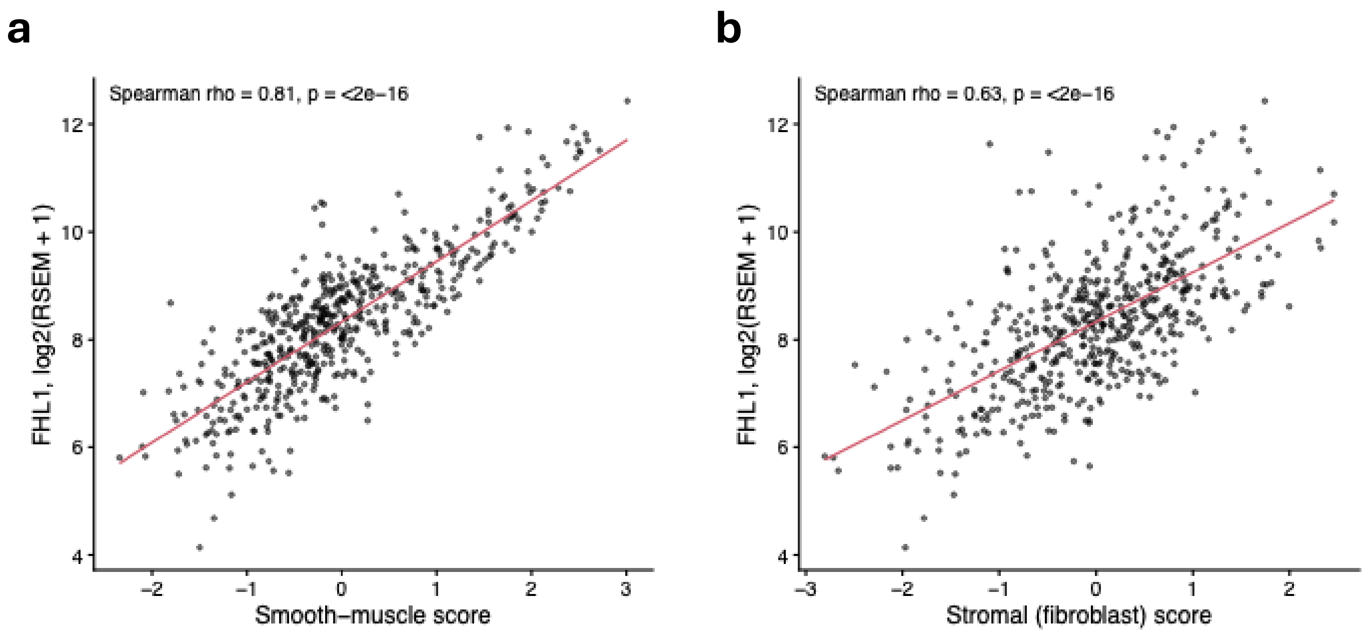

Figure S3. Correlation of bulk FHL1 expression (log2 RSEM + 1) with (a) the smooth-muscle score and (b) the stromal fibroblast score in the TCGA CRC cohort. Spearman correlation coefficients and P values are shown.

Figure S4

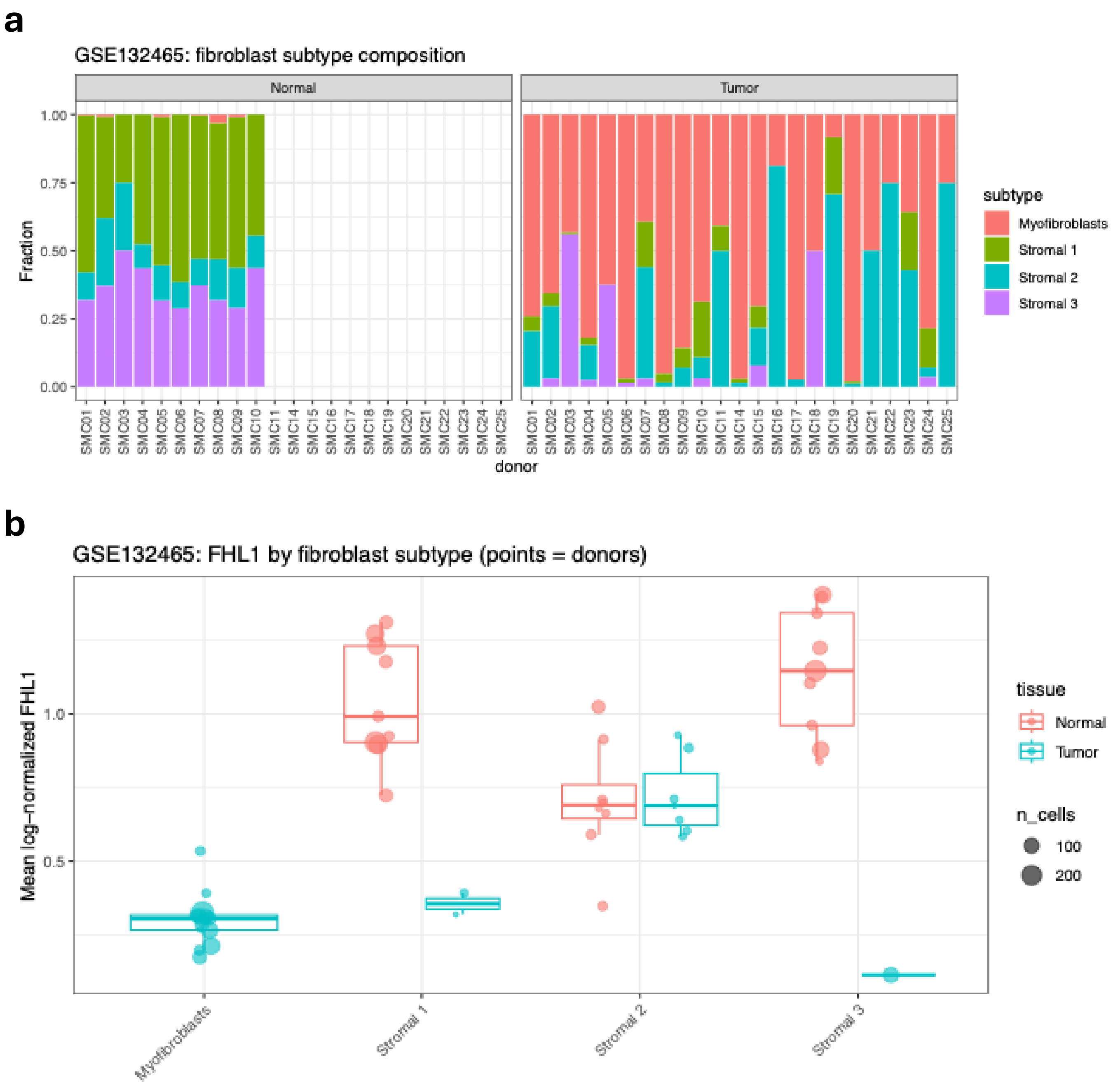

Figure S4. Fibroblast subtypes in GSE132465. (a) Fibroblast subtype composition (Stromal 1, Stromal 2, Stromal 3, myofibroblasts) in normal and tumor tissue of each patient. (b) Mean log-normalized FHL1 expression by fibroblast subtype in normal and tumor tissue; each point is one patient, and point size indicates the number of cells.

Figure S5

a

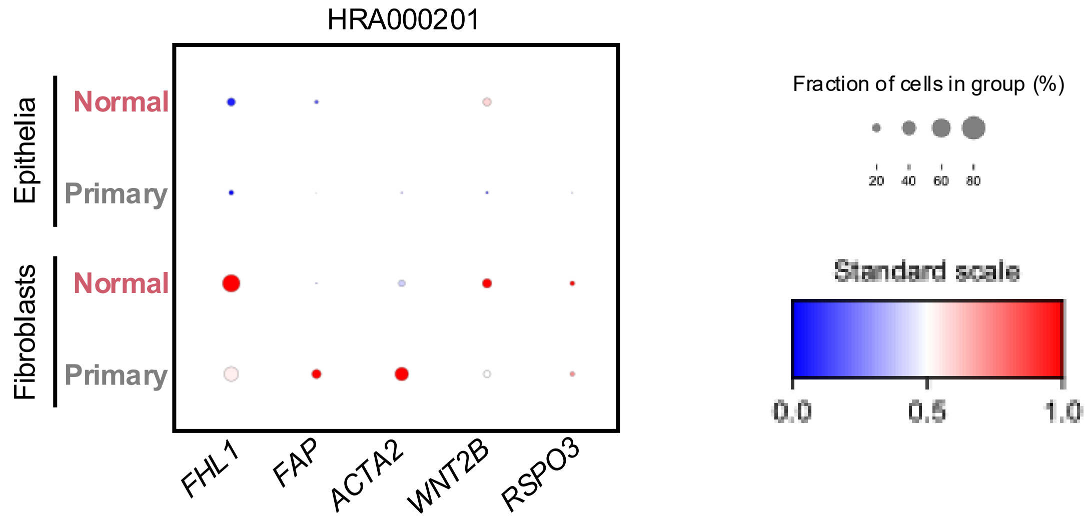

Figure S5. Dot plot of FHL1, FAP, ACTA2, WNT2B and RSPO3 in epithelial cells and fibroblasts from normal and primary tumor tissue in HRA000201. Dot size indicates the fraction of cells expressing each gene; color indicates standardized mean expression.

Figure S6

a

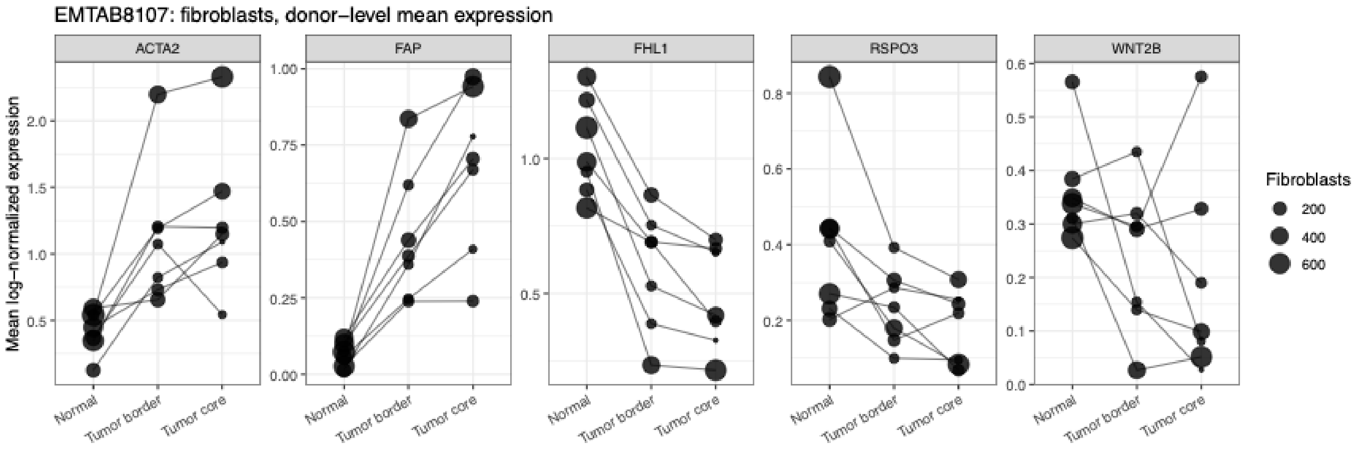

b

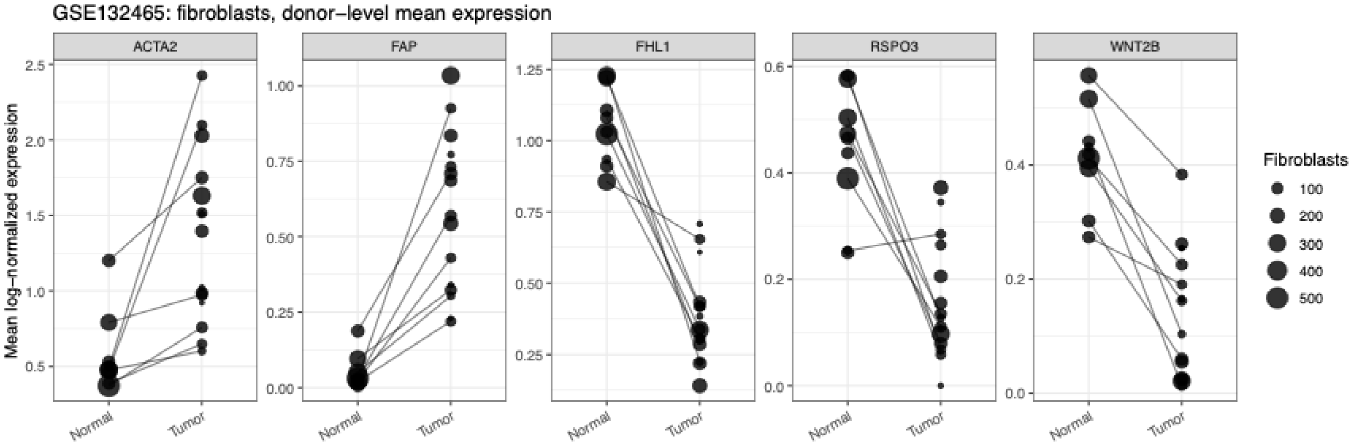

Figure S6. Donor-level mean expression (log-normalized) of FHL1, FAP, ACTA2, WNT2B and RSPO3 in fibroblasts from (a) normal tissue, tumor border and tumor core in EMTAB8107 and (b) normal and tumor tissue in GSE132465. Each point is one patient; point size indicates the number of fibroblasts, and lines connect samples from the same patient.
